# Preventing Wernicke Encephalopathy After Bariatric Surgery

**DOI:** 10.1007/s11695-018-3262-4

**Published:** 2018-04-24

**Authors:** Erik Oudman, Jan W. Wijnia, Mirjam van Dam, Laser Ulas Biter, Albert Postma

**Affiliations:** 1 Experimental Psychology, Helmholtz Institute, Utrecht University, Heidelberglaan 1, 3584 CS Utrecht, The Netherlands; 2 Korsakoff Center Slingedael, Lelie Care Group, Rotterdam, The Netherlands; 3 Department of Bariatric Surgery, Franciscus Gasthuis, Rotterdam, The Netherlands

**Keywords:** Clinical nutrition, Dietary, Bariatric, Gastric, Obesity, Wernicke’s encephalopathy, Thiamine

## Abstract

Half a million bariatric procedures are performed annually worldwide. Our aim was to review the signs and symptoms of Wernicke’s encephalopathy (WE) after bariatric surgery. We included 118 WE cases. Descriptions involved gastric bypass (52%), but also newer procedures like the gastric sleeve. Bariatric WE patients were younger (median = 33 years) than those in a recent meta-analysis of medical procedures (mean = 39.5 years), and often presented with vomiting (87.3%), ataxia (84.7%), altered mental status (76.3%), and eye movement disorder (73.7%). Younger age seemed to protect against mental alterations and higher BMI against eye movement disorders. The WE treatment was often insufficient, specifically ignoring low parenteral thiamine levels (77.2%). In case of suspicion, thiamine levels should be tested and treated adequately with parenteral thiamine supplementation.

## Introduction

The prevalence of morbid obesity has risen to global epidemic proportions and bariatric surgery has been shown to be the most effective treatment to achieve substantial and long-lasting weight loss for morbid obesity [1–3]. In the past decades, the number of bariatric procedures performed has increased exponentially. Currently, laparoscopic Roux-en-Y gastric bypass and laparoscopic sleeve gastrectomy are the most commonly performed bariatric procedures with more than 500,000 interventions worldwide per year [4–6]. Wernicke’s encephalopathy (WE) is an acute neuropsychiatric syndrome resulting from malnutrition and a possible adverse complication from bariatric operations. WE is characterized by the classic triad of ataxia, eye movement disorders, and mental status change. The prevalence rate of WE is 0.6–2% of the population, but the condition is often only discovered at autopsy [7]. Current guidelines for bariatric surgery suggest preventive thiamine suppletion (12 mg) in multivitamin treatment for all patients undergoing surgery, but higher doses for patients with suspicion for deficiency [8]. The aim of this paper is to review the clinical characteristics of WE after bariatric surgery, also referred to as “bariatric beriberi” [9] and to raise the clinician’s index of suspicion about this neuropsychiatric diagnosis and its preventability.

## Methods

We searched MEDLINE, EMBASE, and Google Scholar, using MeSH terms (WE, Korsakoff syndrome, beriberi, restrictive weight loss surgery, gastrectomy). There were no language restrictions. Studies published from 1985 to 2017 on bariatric surgery with a diagnosis of WE were included. We reviewed the title and abstract of these articles, and indexed the data for year of publication, age, sex, BMI, onset duration and progression of symptoms, radiographic findings, treatment, and follow-up. All included studies were either case reports or case series, since information on the course of illness and symptomatology was often lacking in all group studies. The maximum number of represented case descriptions in one study was five [10]. One study reviewed four cases [11], three studies reviewed three cases [12–14], and eight cases reviewed two cases [15–23]. Cases were excluded if too little information was available to confirm a diagnosis of WE or no clinical characteristics regarding the patient or course of illness were available. Since the collected data is not a random sample of cases, and not likely to be normally distributed, nonparametric statistical procedures were applied (Mann-Whitney *U* test for comparison of two independent means, chi-square test for multiple means). The recorded data are either number of patients (percentage) or median (range) as appropriate.

## Results

### General Overview

**Fig. 1:**
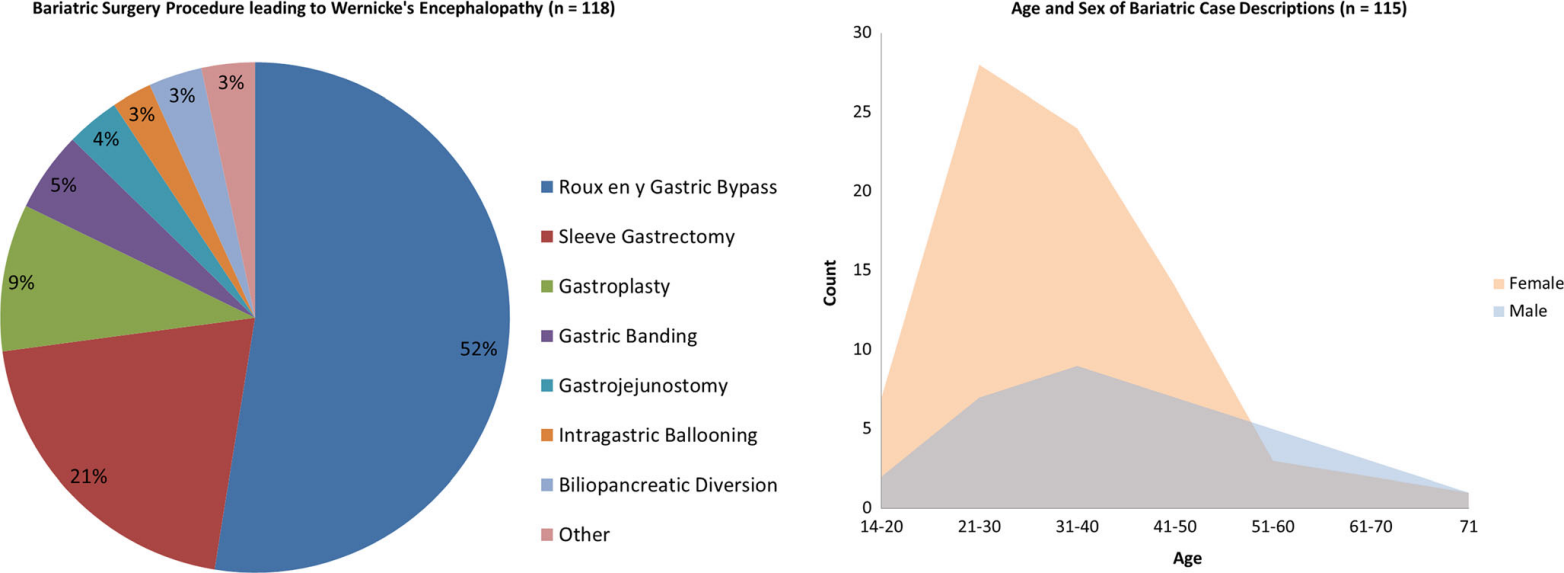
Bariatric procedure case descriptions (*n* = 118) leading to Wernicke’s encephalopathy (left), gender and age distribution of case descriptions on Wernicke’s encephalopathy after bariatric surgery (right, *n* = 113)

We identified 118 case descriptions in the published literature [9–101]. The most common bariatric procedure was Roux-en-Y gastric bypass [9–13, 15–18, 24–63], followed by sleeve gastrectomy [19, 64–85] (see Fig. 1 for an overview on the characteristics of the identified bariatric cases that subsequently developed WE).

**Fig. 2:**
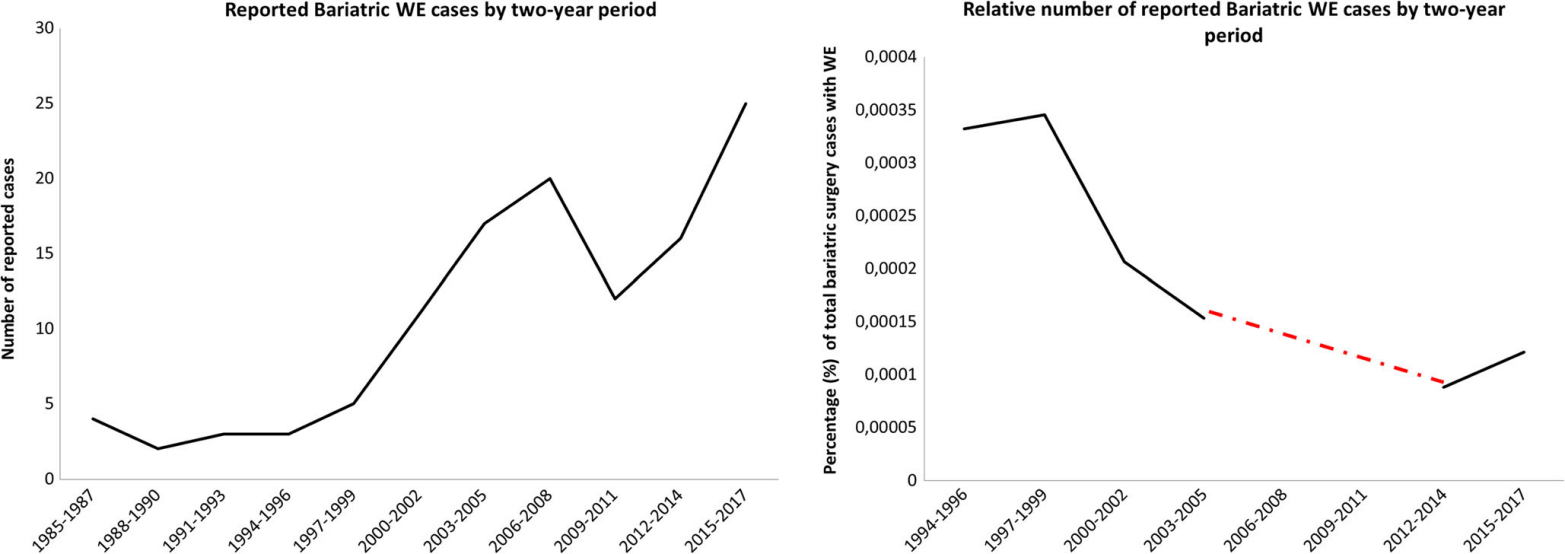
Reported bariatric WE cases by 2-year period (left) and relative reported WE cases by 2-year period compared to general reference information from NHDS and NSAS databases (1993–2006) [23] and ASMBS (2011–2016) [102]. The red dotted line represents missing information

Importantly, new cases of WE have continuously been published since the early beginning of weight loss surgery, and the total number of reported bariatric WE cases is growing per 2-year period (Fig. 2), suggesting that it is still relevant to review this differential diagnosis. Also, the total number of bariatric interventions (NHDS and NSAS databases (1993–2006) [102] and ASMBS database (2011–2016) [103]) has been rising each year [5], resulting in a relative decrease of WE cases per intervention (Fig. 2).

**Fig. 3:**
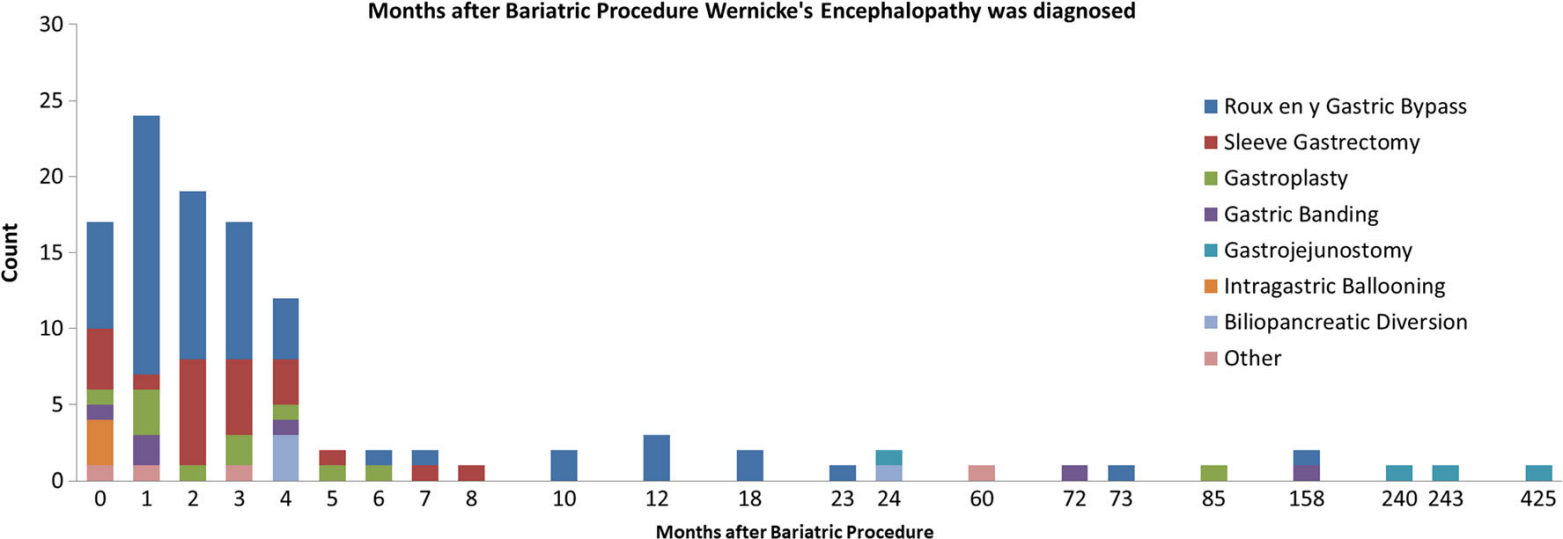
Months after bariatric procedure, Wernicke’s encephalopathy was diagnosed per surgical procedure (*n* = 115)

Descriptions of sleeve gastrectomy [19, 64–85] had a more recent publishing date (median 2014) than papers on Roux-en-Y gastric bypass [9–13, 15–18, 24–63] (median 2006) (*U* (85) = 301.5, *p* < .001), possibly reflecting the increasing popularity of sleeve gastrectomy in the last few years as the bariatric treatment of choice. Furthermore, the gastroplasty cases [20, 86–94] were older (median 1997) than the Roux-en-Y gastric bypass cases [9–13, 15–18, 24–63] (*U* (72) = 96, *p* < .001) and sleeve gastrectomy cases (*U* (34) = 4.6, *p* < .001). The latency between bariatric surgery and onset of WE symptoms was not significantly different between the three most reported surgical procedures (χ^2^ (93) = .299, n.s., median 2–3 months), despite the large surgical differences between the procedures (Fig. 3). All case descriptions on intra gastric ballooning [10, 99, 100] developed WE in the first month post-procedure, while reports on obsolete procedures [21, 22, 99] were up until 24 months following the bariatric procedure. WE developed in the first month after bariatric surgery (17 cases) until the 425th month after surgery (1 case), suggesting a broad range of onset. A large majority of 89 cases (79.1%) developed WE in the first 6 months after surgery. A trend effect suggests that WE tends to develop earlier post-surgery in females (median 2 months post-surgery) than that in males (median 4 months post-surgery) (*U* (109) = 1031.5, *p* = .09, see Fig. 1).

### Vomiting

We further analyzed the symptomatology in all case descriptions. Vomiting was the most frequently described presenting symptom (103 cases, 87.3%) and could be seen as the most relevant precursor of WE. From the literature, it is known that vomiting can also be a major complication in bariatric surgery and is one of the most frequent causes of postoperative readmissions [104]. Severe vomiting is not a normal situation after bariatric surgery and therefore further investigation in cases with frequent vomiting is indicated. In the present sample, non-vomiting cases were distributed throughout all onsets post-surgery, but only 5 out of 15 case descriptions were after the first year, suggesting that other causes than vomiting are likely to cause WE later post-surgery. Alcohol abuse (2 cases), a malabsorptive bariatric procedure (2 cases), and a new operation for hernia (1 case) could explain the late onset in non-vomiting WE presentations, suggesting other factors that negatively affected vitamin B_1_ storage. Importantly, severe infections, such as postoperative intra-abdominal abscesses leading to thiamine deficiency [78], are also a common presenting feature of WE and are likely to relate to an adverse outcome of WE [105].

### Wernicke Encephalopathy: Presenting Characteristics

The most profound characteristic of WE in the reviewed case descriptions was ataxia (84.7%, 100 cases), presenting itself as gait abnormalities up to the full inability to walk or move. The second characteristic was an altered mental status (76.3%, 90 cases), presenting itself as delirium, confusion, and problems in alertness or cognition. The third characteristic was eye movement disorders (73.7%, 87 cases), such as nystagmus and ophthalmoplegia, resulting from extraocular muscle weakness. The full triad was present in 54.2% (64 cases), a percentage much higher than the originally reported 16% of patients that present themselves with the full triad in literature in post-mortem case descriptions of WE in alcoholics [105].

Post hoc analysis in the reviewed sample shows that patients presenting themselves with mental status change were older (median 36 years) than patients without mental status change (median 25.5 years) (*U* (66) = 262, *p* < .005), suggesting that mental status change due to WE is more likely to occur in older bariatric patients. Importantly, this finding sheds new light on young bariatric cases with WE, suggesting relatively lower susceptibility to confusion, disorientation, or problems regarding alertness or consciousness in this specific patient population in their teens and twenties.

Moreover, patients with eye movement disorders had a lower BMI (median 45.6 kg/m^2^) than patients without eye movement disorders (median 52.1 kg/m^2^), suggesting that a higher BMI can protect against this symptom of WE in bariatric cases. Male patients that did not present themselves with eye movement disorders had a later onset of symptoms (median 24.0) than male patients that did have eye movement disorders (median 3.5) (*U* (33) = 49, *p* < .05), suggesting that eye movement disorders are less likely in later phases after bariatric surgery for males than in the acute phase. Possible mechanisms of action causing such effects are elaborated on in our discussion.

### Imaging

CT scans of the brain did not reveal any significant radiological finding in all cases undergoing this procedure (13 cases), suggesting that CT imaging is not the most suitable imaging technique to detect WE. In 65.6% of the case descriptions where an MRI was performed (40 cases) the procedure revealed radiological alterations. This percentage is somewhat higher than the reported sensitivity of 53% in an earlier study on WE [106]. Of interest, positive MRI results were more frequently associated with mental status change (χ^2^ (1) = 3.9, *p* < .005), but not with ataxia (χ^2^ (1) = 1.1, *p* = n.s.) or eye movement disorders (χ^2^ (1) = 0, *p* = n.s.).

### Treatment: Too Little Too Late

According to the European Federation of Neurological Societies and the Royal College of Physicians, 500 mg of parenteral thiamine should be given three times daily until symptoms of acute WE resolute. The treatment is lifesaving and has the potential to reverse this acute neuropsychiatric syndrome [107]. A total of 57 (47.5%) case descriptions were reported in detail on the treatment of WE symptoms. Suboptimal treatment, with relatively low doses of parenteral thiamine (< 500 mg/day), was relatively common (77.2%, 44 cases).

**Fig. 4:**
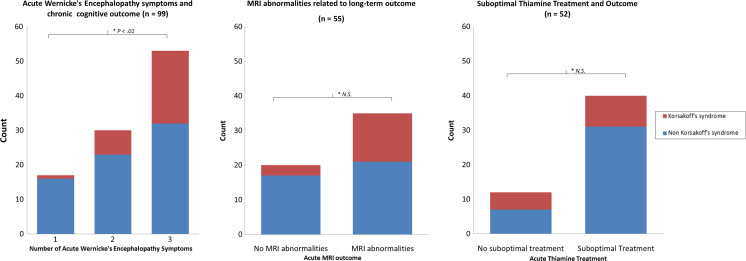
Long-term cognitive outcome related to number of acute symptoms (left), MRI outcome (middle, *n* = 55), and too low levels of thiamine treatment (right, *n* = 52)

Importantly, a progressive clinical course was visible in 31.6% of the patients (37 cases), resulting in post-acute deterioration of neuropsychiatric and neurological symptoms. This suggests that the diagnosis was easily missed, resulting in a lower likelihood of full recovery. Moreover, the detrimental effect of not treating WE promptly is visible in Fig. 4 showing that many of the patients who developed more than one acute symptom later progressed into chronic Korsakoff’s syndrome. This neuropsychiatric disorder is characterized by severe amnesia, executive problems, and confabulations, leading to lifelong impairment [108]. Patients that developed Korsakoff’s syndrome had significantly more acute symptoms (median 3 symptoms) than patients that did not develop Korsakoff’s syndrome (median 2 symptoms) (U (99) = 703.5, *p* < .01). These results highlight the importance of adequate treatment of WE in bariatric patients with high doses of thiamine, to prevent patients from chronic amnesia.

Although this finding was not significant, in the group that presented themselves with acute MRI abnormalities, more cases later developed Korsakoff’s syndrome (Fig. 4). Also, too low dose of a dose of thiamine suppletion therapy resulted in more cases of KS despite the lack of significance.

### Non-compliance

Of interest, in 10.3% of the case descriptions (12 cases), non-compliance to the medication and follow-up medical regimen was reported. A lack of insight into a given situation is a relatively common sign of the acute and chronic phase of WE [105]. The patients did not follow their follow-up, did not take prescribed drugs, or discharged themselves from the hospital against advice, leading to adverse outcomes. Because of the severity of the syndrome, this aspect requires specific attention in the treatment of WE patients, and at risk bariatric patients.

## Discussion

Persistent vomiting is a common symptom suggesting a complication after bariatric surgery [109]. Nausea, vomiting, and a loss of appetite are also common, non-specific symptoms of thiamine deficiency [8]. Ultimately, vomiting and a loss of appetite are also a preventable cause of thiamine deficiency [110], leading to Wernicke’s encephalopathy (WE) in the majority of bariatric case reports. Adequate, timely, prophylactic, and substantial thiamine treatment in all patients undergoing bariatric surgery is required to prevent the development of WE, which is a rare but severe complication. The present review highlights that current treatment was neither prophylactic, adequate, timely, nor substantial in the majority of cases, leading to worsening of WE symptoms, the development of additional WE symptoms, and ultimately chronic Korsakoff’s syndrome.

One of the most remarkable findings in the present review is that the initial symptoms of WE are often not recognized as such, leading to a prolonged state of emergent WE. In 31.6% of the cases, the initial symptoms progressed into more severe symptoms, ultimately leading to chronic Korsakoff’s syndrome. Prompt treatment of the first symptoms suggestive of WE with high doses of parenteral thiamine replacement therapy is necessary to prevent further damage [110]. According to the European Federation of Neurological Societies and the Royal College of Physicians, 500 mg of parenteral thiamine should be given three times daily until symptoms of acute WE resolve [107]. Interestingly, guidelines for treating WE suggest that patients suspected of WE should already be treated as such [107, 111]. Additionally, prophylaxis of WE following early signs and symptoms is only achieved by use of parenteral vitamin supplements, since oral supplements are not absorbed in significant amounts [111]. Moreover, in bariatric surgery, it is always relevant to give prophylactic vitamin therapy, according to international guidelines, to prevent patients from WE.

Of interest, newer methods for bariatric surgery such as sleeve gastrectomy and intragastric ballooning still can lead to WE, despite their relative benefits for the patient. Recently, Armstrong–Javors (2016) pointed out that new techniques lead to the primary risk factor of WE, namely vomiting, despite a theoretical advantage by reducing the stomach volume without bypassing the duodenum [112]. Suspicion for WE should therefore be equally high in more traditional surgical procedures and newer procedures. Also, the risk of developing WE due to vitamin B_1_ deficiency is not restricted to the first half year after surgery but appears to be lifelong, given other factors such as new infections, insufficient meals, or alcohol consumption [110, 113, 114]. Preventive education on the necessity of sufficient vitamin intake should be given before bariatric surgery is performed and is relevant in long-term follow-up.

Bariatric patients in their teens or twenties are likely to be more protected for mental status change in the course of WE than patients in their thirties or older, as reflected in a younger age of non-mental status change patients. This finding is in line with earlier reports showing that age is the strongest predictor for postoperative delirium [115, 116]. Importantly, pediatric patients and young adults undergoing bariatric surgery therefore require more attention for sensorimotor problems, such as ataxia and eye movement disorders, besides prophylactic parenteral thiamine treatment. In this specific group, more attention to lifestyle training should be an essential element of treatment, since non-compliance is relatively higher [50]. Relatively more cognitive reserve in combination with non-compliance can leave symptoms of WE unnoticed for a longer period.

Although eye movement disorders such as nystagmus and ophthalmoplegia were much more common in bariatric cases than those in the general WE population [113], a higher preoperational BMI was predictive for fewer eye movement disorders. Additionally, male subjects with longer post-bariatric onsets often had no eye movement disorders as a presenting characteristic of WE. It is likely that eye movement disorders represent the most severe form of thiamine deficiency, since it is also the least common phenomenon of the WE triad. Moreover, females are at greater risk for full thiamine depletion than males [8]. A possible mechanism of action explaining the protective effect of higher weight is a greater storing reserve of thiamine in severely obese patients in comparison with less severely obese patients. This mechanism of action has been referred to as “preferential intracellular thiamine recycling” [116], leading to relatively less thiamine depletion in patients with higher body weight. Often, cases with WE following anorexia nervosa present themselves first with eye movement disorders [117], suggesting that this symptom is likely to be the result of full thiamine depletion. This suggests that both patients with lower body weight, and female patients are at greater risk for developing WE, and should guide clinicians in preventive thiamine therapy [1–4, 118].

Radiologic imaging can be employed to support the diagnosis of WE, but is not always sensitive to WE symptomatology. Often, hyperintensities were visible in the thalamic region, the mammillary bodies, and the region around the third and fourth ventricle, in line with previous research on WE [7]. Our results show that MRI alterations are frequently associated with mental status change, but not the motoric aspects of WE. This finding is relevant, because it suggests that specifically in bariatric patients with motoric problems, such as ataxia or eye movement disorders, WE should be treated despite the outcome of an MRI.

Non-compliance is common in WE patients following bariatric surgery (10.3%) and could be viewed as a more discrete symptom of the disorder. Patients with WE lack insight into their situation, due to the severity of the neurological problems [108, 110]. Education on the direct adverse consequences of malnourishment should be incorporated into the provision of information before surgery. After surgery, more automated checks on vomiting are relevant.

A limitation of the present review is that we only reviewed case descriptions. Therefore, predictive information regarding prevalence rates and incidence rates is limited. Despite this limitation, the level of detail in the reviewed case studies leads to new insights into WE following bariatric surgery.

Recently published studies on treatment perspectives of WE in general and psychiatric hospitals are alarming: European as well as American studies demonstrated that most patients did not receive thiamine at all or only received it orally in low doses [119, 120]. Both types of treatment lead to unnecessary cases of chronic Korsakoff’s syndrome characterized by severe amnesia, executive problems, and confabulations, leading to lifelong impairment [108]. It is therefore important to highlight the clinical signs of symptoms in this specific condition.

In conclusion, there is a growing number of bariatric patients worldwide. Malnourishment-related WE is a rare but severe and preventable consequence of bariatric surgery that warrants attention given its rapid onset and detrimental course. All bariatric procedures can lead to deficiencies and therefore to WE. WE can be fully prevented by supplying prophylactic thiamine given either parenterally in vomiting patients or orally in non-vomiting patients. Mental confusion, eye movement disorders, and ataxia are often missed as crucial symptoms of WE. After the initial onset of symptoms, rapid treatment with high doses of thiamine is still a life-saving measure, directly ameliorating the core symptoms of WE. The large distribution of WE onsets suggests that bariatric patients remain more vulnerable to vitamin B_1_ deficiency for life, and therefore require lifelong routine follow-up on their B_1_ status.
